# Potential Anticancer Mechanisms of a Novel EGFR/DNA-Targeting Combi-Molecule (JDF12) against DU145 Prostate Cancer Cells: An iTRAQ-Based Proteomic Analysis

**DOI:** 10.1155/2017/8050313

**Published:** 2017-10-15

**Authors:** Haofeng Zheng, Guancan Liang, Yanxiong Chen, Sijie Lin, Wei Liu, Youqiang Fang

**Affiliations:** ^1^Department of Urology, The Third Affiliated Hospital of Sun Yat-Sen University, Guangzhou 510630, China; ^2^Guangdong Provincial Key Laboratory of Liver Disease Research, The Third Affiliated Hospital of Sun Yat-Sen University, Guangzhou 510630, China

## Abstract

The development of multitargeting drugs is an emerging trend in cancer research. To promote further development and clinical application of multitargeting drugs, this research was performed. MTT assay and flow cytometry of Annexin V/propidium iodide staining were used to confirm the proapoptotic efficacy of a novel combi-targeting molecule, JDF12, against DU145 prostate cancer (PCa) cells. Differentially expressed proteins between control and JDF12-treated cultures were revealed by isobaric tags for relative and absolute quantitation (iTRAQ), and part of them was confirmed by quantitative PCR. Differentially expressed proteins were further analyzed for function, pathway association, and protein−protein interactions using GO, KEGG, and STRING databases. A total of 119 differentially expressed proteins, 70 upregulated and 49 downregulated, were implicated in the anticancer effects of JDF12. Many of these proteins are involved in biosynthesis, response to stress, energy metabolism, and signal transduction. This study provides important information for understanding the anti-PCa mechanisms of JDF12, and well-designed combi-targeting drugs may possess stronger anticancer efficacy than single-targeting drugs and are thus promising candidates for clinical application.

## 1. Introduction

Prostate cancer (PCa) is one of the most commonly diagnosed solid organ malignancies and remains the third leading cause of cancer death among men in the United States [1]. It is estimated that more than 161,000 new PCa diagnoses and over 26,000 deaths will occur in America during 2017 [2]. Metastatic castration-resistant prostate cancer (mCRPC) is the end stage of PCa, and often leads to death within two years [3].

While many therapies are initially effective, recurrence and treatment failure are common. Acquired drug resistance and other changes in the biological behavior of cancer cells are major impediments to long-term control or cure [4, 5]. Joint use of multiplex drugs may lessen drug resistance, but serious drugs toxicities have been reported [6]. In light of these problems, development of multitargeting drugs is one promising alternative [7]. In our previous studies, we developed a combi-targeting molecule, JDF12, with both antiepidermal growth factor receptor (EGFR) and DNA-alkylating properties. In situ, JDF12 is hydrolyzed to JDF04R, which can inhibit the phosphorylation of EGFR and activation of isolated EGFR tyrosine kinase. In addition, JDF12 is hydrolyzed to a DNA-alkylating agent [8]. Subsequent studies showed that JDF12 exhibited not only stronger anticancer effects than single drugs or joint use of two drugs at equivalent doses, but also better toxicity profiles and lower drug resistance rate [9, 10].

Although the anticancer effects of JDF12 are well described, the detailed molecular mechanisms of its anticancer efficacy are incompletely understood, preventing further clinical applications. The current study was designed to identify the potential anticancer mechanisms of JDF12 and assess the potential of this combi-targeting drug for anticancer therapy.

## 2. Materials and Methods

### 2.1. Drug Treatment

The combi-targeting drug JDF12 was synthesized as described in our previous study [9]. The drug was kept at −20°C and dissolved in dimethyl sulfoxide (DMSO) for in vitro application. Fetal bovine serum (FBS, 10%) was used as a diluent so that the final DMSO concentration was below 0.2%.

### 2.2. Cell Culture

The human PCa cell line DU145, PC3, and 22Rv1 were obtained from the cell bank of the Type Culture Collection of the Chinese Academy of Sciences (Shanghai, China). Cells were cultured in RPMI-1640 medium (Gibco, USA) supplemented with 10% FBS (PAN, Germany) and maintained at 37°C in a humidified incubator under a 5% CO_2_/95% air atmosphere. Cells were subcultured every 2-3 days as previously described [9].

### 2.3. Cell Viability

Cells in log-phase were plated at 5 × 10^3^/well in 96-well plates for 24 h. Cells were then treated with a range of JDF12 concentrations for 48 h. An MTT kit (KeyGEN BioTECH, Jiangsu, China) was used to determine cell viability according to the manufacturer's protocol. Briefly, MTT was added to each well (0.5 mg/ml final concentration) for 4 h following JDF12 treatment. The crystals produced from MTT by viable cells were dissolved in 150 *μ*l DMSO for 15 min and optical density was measured on a microplate reader (BioRab, USA) at 490 nm. The half-maximal inhibitory concentration (IC_50_) of JDF12 was determined from the dose-response curve. In addition, the time course of survival at the IC_50_ was measured over 48 h. Three independent experiments were performed at each concentration.

### 2.4. Flow Cytometry

An Apoptosis Detection Kit (KeyGEN) for Annexin V-FITC and propidium iodide (PI) staining was used to assess cell apoptosis. Briefly, cells treated as described were washed with ice-cold PBS, harvested by trypsinization, and resuspended in binding buffer at 1 × 10^6^ cells/mL. Then, 500 *μ*L cell suspension (approximately 5 × 10^5^ cells) was incubated with 5 *μ*L PI (0.5 mg/mL) and 5 *μ*L Annexin V-FITC for 15 min at 25°C in the dark. A flow cytometer (FACSCalibur, Becton Dickinson, San Jose, CA, USA) with emission at 530 nm for FITC and 630 nm for PI and excitation at 488 nm was used to analyze the proportions of cells in early and late apoptosis. Three independent experiments were performed at each time point.

### 2.5. iTRAQ Proteome Analysis

#### 2.5.1. Protein Extraction

DU145 cells were seeded in 75-cm^2^ flasks (1 × 10^6^ cells) for 24 h and treated with the JDF12 IC_50_ concentration for an additional 24 h. Cells were then washed thoroughly with ice-cold PBS and lysed with RIPA buffer (KeyGEN) according to the manufacturer's instructions. Lysates were centrifuged at 12000 ×g for 20 min at 4°C and the protein concentration of each supernatant sample was measured using a BCA protein assay kit (KeyGEN). The extracted protein solutions were stored at −80°C for later analysis with no repeat freeze-thaw cycles.

#### 2.5.2. Trypsin Digestion and iTRAQ Labeling

The reagents and buffers for isobaric tags for relative and absolute quantitation (iTRAQ) labeling and cleaning were purchased from Applied Biosystems (Foster City, CA, USA). The iTRAQ labeling assay was conducted according to the manufacturer instructions. Briefly, 100 *μ*g of each protein sample was dissolved, alkylated, and digested with trypsin (Promega, Madison, WI, USA). After vacuum freeze-drying, the digested peptides were reconstituted in 50 *μ*L of 0.5 M triethylammonium bicarbonate. Peptides were then processed with an iTRAQ-8plex kit. Each sample was labeled with two tags (blank group: 113, 117; JDF12 group: 115, 119). Finally, all labeled samples were mixed in a single vial and dried using a rotary vacuum concentrator.

#### 2.5.3. High pH Reversed-Phase Fractionation

High pH reversed-phase fractionation was performed using a high-performance liquid chromatography system (Phenomenex columns; Gemini-NX 3u C18 110A; 150 × 2.00 mm). Separation of the labeled peptides was achieved by a linear gradient of mobile phase A (20 mM HCOONH_4_, pH = 10) to mobile phase B (20 mM HCOONH_4_, 80% acetonitrile (ACN), pH = 10). The UV detection wavelengths were 214 nm/280 nm. Depending on the peak and time, fractions were collected every 1 min, for a total of 24 fractions. The fractions were acidified with 50% trifluoroacetic acid and dried by vacuum centrifuge.

#### 2.5.4. Reverse-Phase LC-MS Analysis

Peptide samples were dissolved in buffer (0.1% formic acid, 2% acetonitrile) and centrifuged at 12,000 ×g for 20 min at 4°C. The peptides were eluted with a linear gradient of buffer A (0.1% formic acid) to buffer B (0.1% formic acid, 80% ACN) at a flow rate of 330 nL/min for a total of 60 min. The Q Exactive system was used for MS/MS analysis in information-dependent acquisition mode. Mass spectra were acquired over a scan range of 350 to 1800 *m*/*z* with a resolution of 70,000 using maximum injection time (40 ms) per spectrum. Fragmentation detection used the twenty most intense precursors per MS cycle with 60 ms maximum injection time. Tandem mass spectra were recorded at a resolution of 17,500 with iTRAQ reagent collision energy adjustment “ON” and rolling collision energy “ON.”

#### 2.5.5. Protein Identification and Quantification

Protein identification and quantification results were analyzed by ProteinPilot™ Software 5.0 (AB SCIEX) using the Paragon™ Algorithm (5.0.0.0, 4767). Each MS/MS spectrum was searched against the Uniprot/Swiss-Prot Database for Homo sapiens. Parameters for searching were as follows: (1) Detected Protein Threshold: 0.05; (2) Competitor Error Margin: 2.00; (3) Revision Number: 4769; (4) Instrument: Orbi MS (1−3 ppm), Orbi MS/MS; (5) Sample Type: iTRAQ 8 plex (Peptide Labeled); (6) Cysteine Alkylation: MMTS; (7) Digestion: Trypsin; (8) Special Factors: none; (9) ID Focus: biological modifications; (10) Search Effort: thorough ID; (11) FDR Analysis: yes; (12) User Modified Parameter Files: no. Qualification criteria for peptides were unused confidence score ≥ 1.3 and confidence level ≥ 95%. Proteins containing at least one peptide and false discovery rate (FDR) < 1% were accepted. Proteins with poor repeatability (coefficient of variation > 0.5) or no quantitative information were removed. For qualifying proteins, average fold change ≥ 1.5 was classified as upregulated and average fold change ≤ 0.67 was defined as downregulated.

### 2.6. Gene Ontology and KEGG Pathway Enrichment Analysis

The biological functions of the significantly up- or downregulated proteins were analyzed using web-based Gene Ontology (GO) software (http://www.geneontology.org/). There are three main modules in the GO project: biological process, cellular component, and molecular function. Pathway analysis was conducted using by the web-based Kyoto Encyclopedia of Genes and Genomes (KEGG, http://www.kegg.jp/). Hierarchical clustering is presented with java Tree view using Cluster 3.0. Known and predicted protein−protein interaction networks of differentially expressed proteins were built based on the publicly available Search Tool for the Retrieval of Interacting Genes/Proteins (STRING) database (http://string-db.org/). FDR adjusted *p* value of 0.05 was considered statistically significant.

### 2.7. qPCR Analysis

Total RNA was extracted using the TRIzol Reagent (Life Technologies™) according to the manufacturer's protocol. cDNA was synthesized from 1 *μ*g of total RNA using the Transcript of First Strand cDNA Synthesis Kit (Roche). Quantitative (q)PCR was performed on a Light Cycler 480 (Roche) using SYBR Green PCR Master Mix according to the manufacturer's instructions. *β*-Actin was used as the endogenous control to normalize target gene expression. Primers were synthesized by Ruibotech (Beijing, China). Relative RNA expression was calculated by the 2^−ΔΔCT^ method. All samples were measured three times, and results are shown as mean ± standard deviation.

### 2.8. Statistical Analysis

Group means were compared by independent samples *t*-test, with *p* < 0.05 considered statistically significant. SPSS version 19.0 (Chicago, Illinois, USA) was used for all statistical calculations.

## 3. Results

### 3.1. JDF12 Reduces Viable Prostate Cancer Cell Number

**Figure 1:**
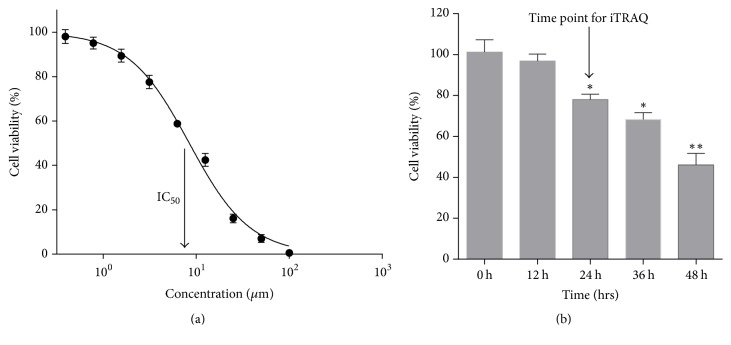
The effects of JDF12 on the cell viability. (a) Concentration course of changes in the cell viability of DU145 cells treated with JDF12 for 48 h; (b) Time course of changes in the cell viability of DU145 cells treated with IC_50_ JDF12. Data was expressed as mean ± SD of 5 determinations from three independent experiments and compared to the blank group (0 h), ^*∗*^*p* < 0.05 and ^*∗∗*^*p* < 0.01.

Human PCa-derived DU145 cells treated with JDF12 from 0.39 to 100.00 *μ*M for 48 h exhibited a progressive decrease in viable cell number as measured by MTT assay, and dose-response curves yielded an average (±SD) IC_50_ value of 8.42 ± 0.40 *μ*M (Figure 1(a)). During application of the IC_50_ dose, no significant reduction in cell number was observed at 12 h, while significant reductions were observed at 24 h or longer (Figure 1(b)). A 24-h treatment time was selected for subsequent apoptosis and proteomics measures. MTT results of 22Rv1 and PC3 cell lines at 48 h are shown in Supplemental Figure 1 in Supplementary Material, available online at https://doi.org/10.1155/2017/8050313.

### 3.2. Induction of PCa Cell Apoptosis by JDF12

**Figure 2:**
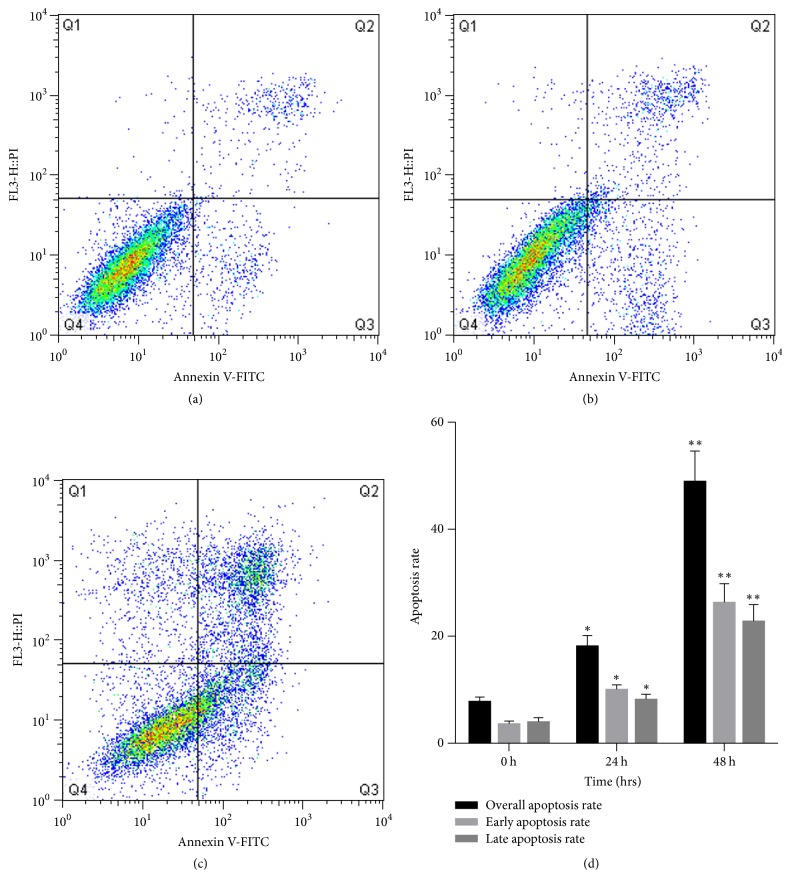
The effects of JDF12 on the cell apoptosis. Early apoptosis cells were Annexin V+/PI−, and later apoptosis cells were Annexin V+/PI+. (a) 0 h; (b) 24 h; (c) 48 h; (d) the overall apoptosis rate was significant increased after treatment of IC_50_ JDF12 for 24 h and 48 h. Results were presented as mean ± SD from three independent experiments. ^*∗*^*p* < 0.05, ^*∗∗*^*p* < 0.01.

Figure 2 shows the apoptosis rates in the blank control and JDF12-treated groups as measured by flow cytometry of Annexin V/PI-stained cells (Annexin V+/PI− indicates early apoptosis and V+/PI+ indicates late apoptosis). Early and late apoptosis rates were summed to yield an overall apoptosis rate for this study. Cell apoptosis rate was significantly increased by 24 and 48 h treatment with JDF12 at the IC_50_ (8.42 *μ*M) compared to the blank group (0 h).

### 3.3. Effects of JDF12 on Protein Expression Levels in PCa Cells

An iTRAQ-based quantitative proteomics approach was used to measure the effects of JDF12 on protein expression levels in DU145 cells. A total of 5610 proteins were detected in the global proteomic analysis with a CV < 50% among replicates. Each protein had at least one identified peptide with an unused score ≥ 1.3, indicating >95% confidence in correct sequence identification. A total of 119 differentially expressed proteins (fold change ≥ 1.5 or ≤0.67) were identified by iTRAQ. Among them, 70 were upregulated and 49 were downregulated. These differentially expressed proteins are summarized in Table 1.

### 3.4. Functional Classification of Differentially Expressed Proteins

**Figure 3:**
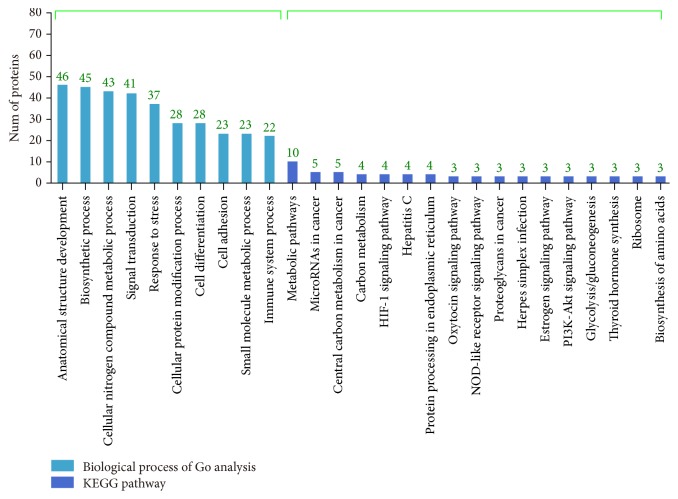
The functional classification of differentially expressed proteins using Go analysis (biological process) and KEGG Pathway. FDR adjusted *p* value of 0.05 was considered statistically significant.

A total of 133 GO terms, including 38 molecular function terms, 66 biological process terms, and 29 cellular component terms, were retrieved. Differentially expressed proteins were further analyzed by KEGG, and 75 proteins were mapped into KEGG pathways. “Metabolic”, “micro-RNA”, and “carbon metabolism” were most affected by JDF12, suggesting that changes in these pathways/processes mediate the anticancer efficacy (Figure 3 and Supplemental Figure 2).

### 3.5. Interaction Analysis of Differentially Expressed Proteins

**Figure 4:**
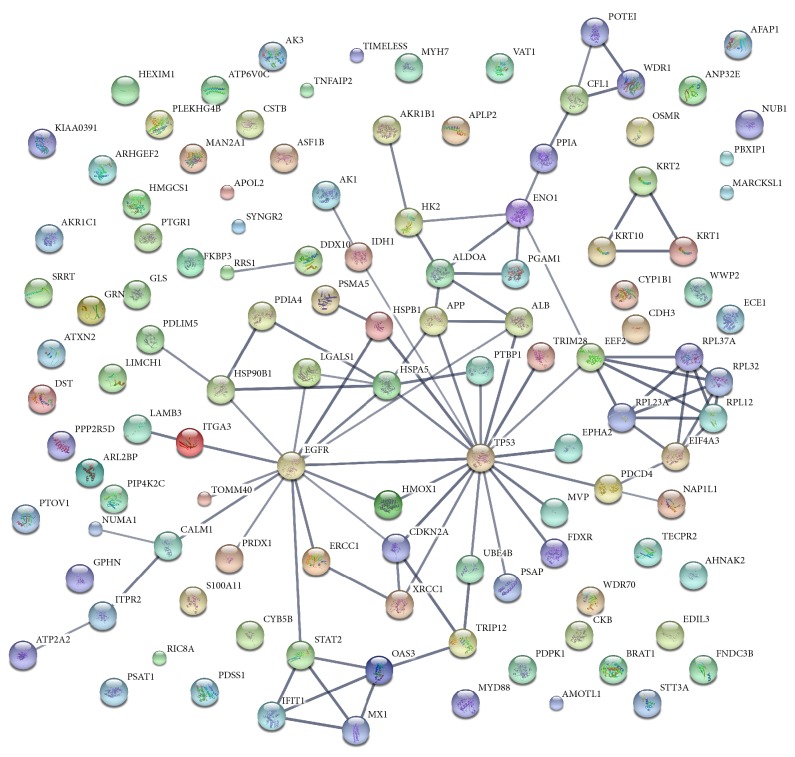
The interaction analysis of differentially expressed proteins using STRING analysis (confidence view). The PPI network score was set to the high level (0.700). Stronger associations are represented by thicker lines. EGFR, TP53, and HSPA5 were identified as “hubs.”

**Figure 5:**
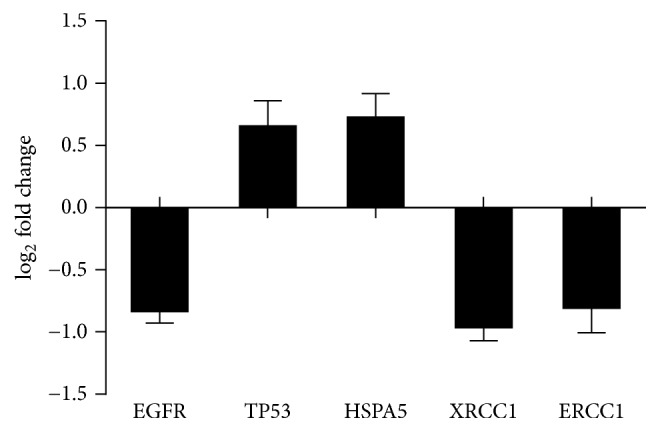
The expression level of some important proteins detected by iTRAQ. Qualification criteria for peptides were unused confidence score ≥ 1.3 and confidence level ≥ 95%. Average fold change ≥ 1.5 was classified as upregulated and average fold change ≤ 0.67 was defined as downregulated.

To identify interactions among differentially expressed proteins, STRING analysis was performed. One hundred and sixteen protein nodes and 90 edges were identified. The epidermal growth factor receptor (EGFR), tumor protein p53 (TP53), and heat shock protein A member 5 (HSPA5) were the top three hubs, indicating highest connectivity and greatest capacity to regulate the interaction network (Figures 4 and 5).

### 3.6. Confirmation of Differential Expression by qPCR

**Figure 6:**
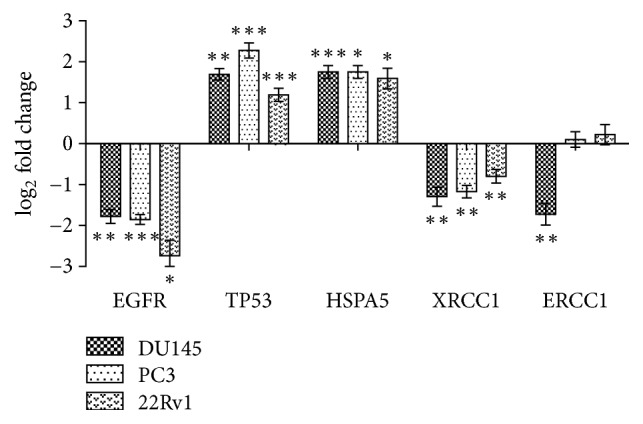
The expression level of mRNA for some important proteins in DU145, PC3, and 22Rv1 cell lines. All samples were measured three times, and results are showed as mean ± standard deviation. Statistical analysis was performed independently in each cell line. ^*∗*^*p* < 0.05, ^*∗∗*^*p* < 0.01, and ^*∗∗∗*^*p* < 0.001.

Quantitative PCR was performed to confirm expression changes of proteins with highest connectivity, including EGFR, TP53, HSPA5, excision repair cross-complementation group 1 (ERCC1), and X-ray repair cross complementing 1 (XRCC1), in several prostate cell lines. The expression levels of all these proteins in DU145 cells were significantly altered, including two upregulated and three downregulated, consistent with iTRAQ. The expressions of these five proteins in other prostate cell lines including PC3 and 22Rv1 were also confirmed by qPCR. All proteins excluding ERCC1 were significantly altered, consistent with results in DU145 cell line. As for ERCC1, no significant difference was found in PC3 and 22Rv1 cell lines (Figure 6).

## 4. Discussion

**Figure 7:**
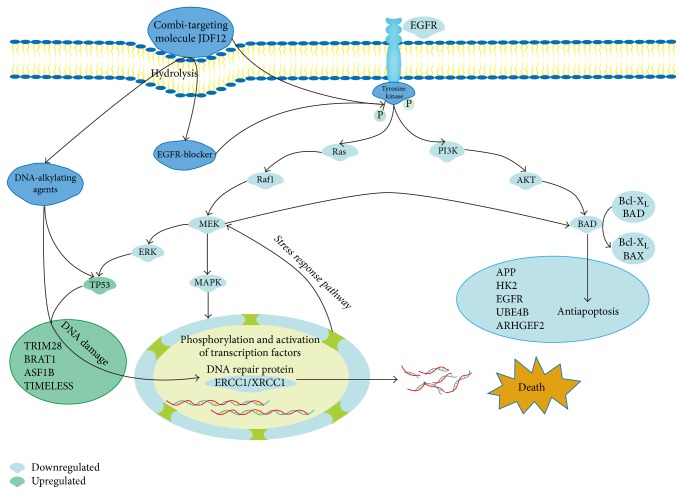
The proposed possible anticancer mechanisms and signaling pathways of JDF12-induced apoptosis in DU145 cells.

This study identified potential molecular mechanisms underlying the anticancer efficacy of the combi-targeting molecule JDF12. Proteomics analysis revealed a myriad of differentially expressed proteins and several signaling pathways strongly linked to JDF12-induced apoptosis, including EGFR and TP53 pathways (Figure 7). These differentially expressed proteins may induce apoptosis of cancer cells by interfering with biosynthesis, response to stress, energy metabolism, and other signal transduction pathways.

Tumors develop resistance to targeted therapies through overexpression of multixenobiotic resistance proteins and rapid replication. Further, hyperproliferation, drug resistance, and metastasis are driven by multiple kinase cascades, and interruption of only one pathway may be insufficient for tumor control. Therefore, single drugs targeting multiple kinases or biological processes (e.g., DNA replication and growth factor transduction) may be required to fully inhibit the growth of cancer cells [11]. Indeed, recent studies have confirmed that the attrition rates of multitargeting agents are lower than single-targeting agents [12]. The initial design concept of JDF12 was to produce an agent with synergistic anticancer effects through inhibition of EGFR transduction and DNA alkylation, and these properties were reconfirmed in this study. We speculate that the EGFR-blocking property may inhibit the activation of DNA repair pathways by DNA alkylation, while DNA alkylation may reduce drug resistance caused by EGFR inhibition.

EGFR signaling pathways are strongly associated with cell survival. Increasing the expression levels of EGFR family proteins, including EGFR, ErbB2, ErbB3, and ErbB4, can promote the growth of cancer cells [13]. Overexpression of EGFR has also been linked to anticancer drug resistance and greater aggression of breast, lung, and other cancers [14–16]. Multiple transduction cascades including the Ras/MAPK pathway are believed to mediate cell survival following EGFR activation [17]. Expression of EGFR was significantly lower in JDF12-treated PCa cells after 24 h, while cells exposed to JDF12 for only 2 h did not show this response [8]. Further, no significant cell death was observed within 12 h. This temporal association suggests that EGFR downregulation by JDF12 may be required to induce apoptosis, although the additional DNA-alkylating effect may also contribute.

The tumor suppressor TP53 is one of the most frequently downregulated proteins in cancers, and many p53 mutants are oncogenic [18]. TP53 contributes to multiple cellular processes associated with cell proliferation and survival, including metabolism, the DNA damage response, senescence, stemness, and differentiation. Among these processes, regulation of the DNA damage response may be the most relevant to cancer [19]. Activation of p53 is the key element in response to DNA damage. ATM (ataxia telangiectasia mutated) and ATR (ataxia telangiectasia- and Rad3-related) are activated by double- or single-strand breaks, which inhibits p53 degradation and leads to transcriptional activation and chromatin remodeling [20]. Moreover, p53 is linked to other proteins involved in apoptosis induction [21]. The expression of TP53 was upregulated by JDF12, suggesting that JDF12 may induce cancer cell apoptosis through TP53 signaling pathways. Indeed, overexpression of TP53 can inhibit the growth of tumors, and again suppression of ERGR signaling may further enhance this proapoptotic effect. Due to the reason that DU-145 cells have mutations in TP53 [22], qPCR was performed to confirm some important changes in other prostate cell lines, including PC3 and 22Rv1. Results suggested that the anticancer effects of JDF12 are generalizable to multiple cell lines, while the detailed mechanisms may be different. Although these three cell lines are all prostate cells, they are at different stages of prostate cancer, and isolated from different tissues, which may contribute to the difference in mRNA expression of ERCC1 among DU145, 22Rv1, and PC3.

HSPA5 is also overexpressed in some cancers, including breast, hepatocellular, and lung cancer [23–25]. Upregulation of HSPA5 promotes drug resistance as well as metastasis, resulting in poor prognosis [26]. It was thus surprising to find that JDF12 induced HSPA5 overexpression, in contrast to many other anticancer drugs. However, the signaling pathways controlling tumor growth are driven by multiple kinases. HSPA5 is also strongly connected to autophagy, although this was not a planned target of JDF12. Upregulation of HSPA5 may be a compensatory response to JDF12. Nonetheless, such a response was insufficient to rescue PCa cells from the DNA-alkylating and EGFR-blocking effects.

Expression levels of ERCC1 and XRCC1, which play important roles in DNA damage repair pathways, were also downregulated by JDF12. Our previous study in nude mice revealed that JDF12 induced DNA damage by inhibiting ERCC1 and XRCC1 expression [9], consistent with the current findings. At the same time, inhibiting the EGFR signaling pathway may contribute to downregulated expression of ERCC1 and XRCC1, thereby augmenting the anticancer effects.

Although the anticancer effects of JDF12 are strong and superior to its prodrugs, the IC_50_ for JDF12 (8.42 uM) is fairly high for an anticancer compound. Some useful compounds have IC_50_'s in the nM range [27]. Differences in kind of drugs, cells, and time of treatment may take main responsibilities for it, but the experimental environment and operator may also have some contributions. All in all, JDF12 could potentially be an effective therapy for prostate cancer.

Combi-targeting drugs are promising anticancer agents, but the therapeutic mechanisms are more complex than those of single-targeting drugs. Indeed, our proteomics analysis revealed that EGFR, TP53, ERCC1, and XRCC1 constitute only a small fraction of the proteins regulated by JDF12 (although KEGG and STRING analyses identified these proteins as critical hubs in the interaction network). Further studies are needed to investigate the effects of the other proteins regulated by JDF12. Moreover, additional studies are needed to assess the anticancer mechanisms of JDF12 in animal models and the anticancer efficacy in patients.

## 5. Conclusions

EGFR and TP53 are critical signaling pathways underlying the anticancer efficacy of JDF12, but additional studies are required to confirm this link as well as to analyze the contributions of other JDF12-regulated proteins and signaling pathways. Nonetheless, this study is the first to assess the anticancer mechanisms of a combi-targeting drug at the cellular and molecular levels, thereby providing a foundation for further development of combi-targeting drugs as cancer therapies. Many current anti-mCRPC drugs inhibit androgen or androgen receptors [28]. Drugs with additional targets, notably EGFR signaling and the DNA damage response, could usher in a new era of anti-mCRPC treatment.

## Supplementary Material

The effects of JDF12 on other cell lines as well as Go analysis of JDF12 on DU145 cells.

## Figures and Tables

**Table 1:** Differentially expressed proteins detected by iTRAQ after being treated with JDF12 in DU145 cells.

Number	Accession number	Gene name	Protein name	Fold change
*Upregulated proteins*				
(1)	Q5T2R2	PDSS1	Decaprenyl-diphosphate synthase subunit 1	34.52
(2)	Q96HN1	PLEKHG4B	PLEKHG4B protein (Fragment)	10.23
(3)	Q14914	PTGR1	Prostaglandin reductase 1	3.78
(4)	P09914	IFIT1	Interferon-induced protein with tetratricopeptide repeats 1	3.46
(5)	A0A0C4DGB6	ALB	Serum albumin	2.47
(6)	C9JIZ6	PSAP	Prosaposin	2.42
(7)	Q53EL6	PDCD4	Programmed cell death protein 4	2.41
(8)	F8W8T1	MX1	Interferon-induced GTP-binding protein Mx1	2.30
(9)	P09382	LGALS1	Galectin-1	2.27
(10)	H0YIV4	NAP1L1	Nucleosome assembly protein 1-like 1	2.17
(11)	P18669	PGAM1	Phosphoglycerate mutase 1	2.14
(12)	P12883	MYH7	Myosin-7	2.11
(13)	P16615	ATP2A2	Sarcoplasmic/endoplasmic reticulum calcium ATPase 2	2.05
(14)	P49006	MARCKSL1	MARCKS-related protein	2.02
(15)	Q6PJG6	BRAT1	BRCA1-associated ATM activator 1	1.97
(16)	Q9Y5A7	NUB1	NEDD8 ultimate buster 1	1.96
(17)	Q03169	TNFAIP2	Tumor necrosis factor alpha-induced protein 2	1.92
(18)	Q99536	VAT1	Synaptic vesicle membrane protein VAT-1 homolog	1.92
(19)	P38919	EIF4A3	Eukaryotic initiation factor 4A-III	1.91
(20)	Q96AQ6	PBXIP1	Pre-B-cell leukemia transcription factor-interacting protein 1	1.89
(21)	Q9UIJ7	AK3	GTP: AMP phosphotransferase AK3, mitochondrial	1.84
(22)	P04080	CSTB	Cystatin-B	1.83
(23)	P12277	CKB	Creatine kinase B-type	1.83
(24)	P62937	PPIA	Peptidyl-prolyl cis-trans isomerase A	1.82
(25)	P52630	STAT2	Signal transducer and activator of transcription 2	1.81
(26)	P14625	HSP90B1	Endoplasmin	1.80
(27)	Q6PJG6	BART1	BRCA1-associated ATM activator 1	1.79
(28)	Q9BTT0	ANP32E	Acidic leucine-rich nuclear phosphoprotein 32 family member E	1.79
(29)	Q01581	HMGCS1	Hydroxymethylglutaryl-CoA synthase, cytoplasmic	1.79
(30)	Q5T9B7	AK1	Adenylate kinase isoenzyme 1	1.78
(31)	J3KQL8	APOL2	Apolipoprotein L2	1.76
(32)	P62750	RPL23A	60S ribosomal protein L23a	1.75
(33)	Q14980	NUMA1	Nuclear mitotic apparatus protein 1	1.73
(34)	P28066	PSMA5	Proteasome subunit alpha type-5	1.72
(35)	P23528	CFL1	Cofilin-1	1.71
(36)	Q8IY63	AMOTL1	Angiomotin-like protein 1	1.71
(37)	Q8TBX8	PIP4K2C	Phosphatidylinositol 5-phosphate 4-kinase type-2 gamma	1.70
(38)	P28799	GRN	Granulins	1.68
(39)	P04792	HSPB1	Heat shock protein beta-1	1.67
(40)	P11021	HSPA5	78 kDa glucose-regulated protein	1.67
(41)	A0A0A0MSZ4	FDXR	NADPH: adrenodoxin oxidoreductase, mitochondrial	1.65
(42)	O94992	HEXIM1	Protein HEXIM1	1.65
(43)	P31949	S100A11	Protein S100-A11	1.64
(44)	Q04828	AKR1C1	Aldo-keto reductase family 1 member C1	1.63
(45)	Q13263	TRIM28	Transcription intermediary factor 1-beta	1.63
(46)	Q8IVF2	AHNAK2	Protein AHNAK2	1.63
(47)	Q9NVP2	ASF1B	Histone chaperone ASF1B	1.63
(48)	O75874	IDH1	Isocitrate dehydrogenase [NADP] cytoplasmic	1.62
(49)	O96008	TOMM40	Mitochondrial import receptor subunit TOM40 homolog	1.61
(50)	P27449	ATP6V0C	V-type proton ATPase 16 kDa proteolipid subunit	1.61
(51)	P06733	ENO1	Alpha-enolase	1.60
(52)	Q00688	FKBP3	Peptidyl-prolyl cis-trans isomerase FKBP3	1.60
(53)	A0A0U1RQC9	TP53	Cellular tumor antigen p53	1.59
(54)	Q9Y6K5	OAS3	2′-5′-Oligoadenylate synthase 3	1.59
(55)	P46977	STT3A	Dolichyl-diphosphooligosaccharide—protein glycosyltransferase subunit STT3A	1.58
(56)	Q9UNS1	TIMELESS	Protein timeless homolog	1.58
(57)	E9PFR3	PPP2R5D	Serine/threonine-protein phosphatase 2A 56 kDa regulatory subunit delta isoform	1.57
(58)	Q14764	MVP	Major vault protein	1.57
(59)	O75083	WDR1	WD repeat-containing protein 1	1.56
(60)	P04075	ALDOA	Fructose-bisphosphate aldolase A	1.56
(61)	P09601	HMOX1	Heme oxygenase 1	1.56
(62)	Q16678	CYP1B1	Cytochrome P450 1B1	1.56
(63)	P62158	CALM1	Calmodulin	1.55
(64)	Q06830	PRDX1	Peroxiredoxin-1	1.55
(65)	P13667	PDIA4	Protein disulfide-isomerase A4	1.54
(66)	P22223	CDH3	Cadherin-3	1.54
(67)	P15121	AKR1B1	Aldose reductase	1.53
(68)	P30050	RPL12	60S ribosomal protein L12	1.53
(69)	Q9BXP5	SRRT	Serrate RNA effector molecule homolog	1.52
(70)	H0Y4G9	MYD88	Myeloid differentiation primary response protein MyD88	1.50
*Downregulated proteins*				
(71)	P13645	KRT10	Keratin, type I cytoskeletal 10	0.27
(72)	O95155	UBE4B	Ubiquitin conjugation factor E4 B	0.38
(73)	Q9BYX7	POTEKP	Putative beta-actin-like protein 3	0.39
(74)	P26599	PTBP1	Polypyrimidine tract-binding protein 1	0.44
(75)	P52789	HK2	Hexokinase-2	0.44
(76)	Q53EP0	FNDC3B	Fibronectin type III domain-containing protein 3B	0.44
(77)	P35908	KRT2	Keratin, type II cytoskeletal 2 epidermal	0.45
(78)	P04264	KRT1	Keratin, type II cytoskeletal 1	0.45
(79)	O43854	EDIL3	EGF-like repeat and discoidin I-like domain-containing protein 3	0.46
(80)	F5H039	GPHN	Gephyrin	0.48
(81)	F8W727	RPL32	60S ribosomal protein L32	0.50
(82)	O43760	SYNGR2	Synaptogyrin-2	0.50
(83)	P05067	APP	Amyloid beta A4 protein	0.50
(84)	Q14669	TRIP12	E3 ubiquitin-protein ligase TRIP12	0.51
(85)	P29317	EPHA2	Ephrin type-A receptor 2	0.51
(86)	F5H8D7	XRCC1	DNA repair protein XRCC1	0.51
(87)	Q9Y4B5	MTCL1	Microtubule cross-linking factor 1	0.53
(88)	Q13206	DDX10	Probable ATP-dependent RNA helicase DDX10	0.54
(89)	P61513	RPL37A	60S ribosomal protein L37a	0.55
(90)	Q9NW82	WDR70	WD repeat-containing protein 70	0.56
(91)	P00533	EGFR	Epidermal growth factor receptor	0.56
(92)	Q8N556	AFAP1	Actin filament-associated protein 1	0.56
(93)	J3KNF8	CYB5B	Cytochrome b5 type B	0.56
(94)	O15040	TECPR2	Tectonin beta-propeller repeat-containing protein 2	0.56
(95)	O94925	GLS	Glutaminase kidney isoform, mitochondria	0.57
(96)	Q8N726	CDKN2A	Tumor suppressor ARF	0.58
(97)	Q99650	OSMR	Oncostatin-M-specific receptor subunit beta	0.58
(98)	P07992	ERCC1	DNA excision repair protein ERCC-1	0.58
(99)	O15091	KIAA0391	Mitochondrial ribonuclease P protein 3	0.60
(100)	Q9NPQ8	RIC8A	Synembryn-A	0.60
(101)	Q9Y617	PSAT1	Phosphoserine aminotransferase	0.60
(102)	Q03001	DST	Dystonin	0.61
(103)	Q13751	LAMB3	Laminin subunit beta-3	0.61
(104)	Q9UPQ0	LIMCH1	LIM and calponin homology domains-containing protein 1	0.61
(105)	O00308	WWP2	NEDD4-like E3 ubiquitin-protein ligase WWP2	0.62
(106)	P13639	EEF2	Elongation factor 2	0.63
(107)	P42892	ECE1	Endothelin-converting enzyme 1	0.63
(108)	Q14571	ITPR2	Inositol 1,4,5-trisphosphate receptor type 2	0.63
(109)	E9PER6	PDPK1	3-Phosphoinositide-dependent protein kinase 1	0.64
(110)	Q92974	ARHGEF2	Rho guanine nucleotide exchange factor 2	0.65
(111)	P26006	ITGA3	Integrin alpha-3	0.66
(112)	Q06481	APLP2	Amyloid-like protein 2	0.66
(113)	Q15050	RRS1	Ribosome biogenesis regulatory protein homolog	0.66
(114)	Q15397	PUM3	Pumilio homolog 3	0.66
(115)	Q99700	ATXN2	Ataxin-2	0.66
(116)	Q16706	MAN2A1	Alpha-mannosidase 2	0.67
(117)	Q96HC4	PDLIM5	PDZ and LIM domain protein 5	0.67
(118)	P49643	PRIM2	DNA primase large subunit	0.67
(119)	Q86YD1	PTOV1	Prostate tumor-overexpressed gene 1 protein	0.67
