# Supplementary material for: Potential Anticancer Mechanisms of a Novel EGFR/DNA-Targeting Combi-Molecule (JDF12) against DU145 Prostate Cancer Cells: An iTRAQ-Based Proteomic Analysis
Source: Biomed Res Int. 2017 Oct 15;2017:8050313. doi: 10.1155/2017/8050313 (PMC5661095; doi:10.1155/2017/8050313)
Supplement: Supplementary file 1 — The effects of JDF12 on other cell lines as well as Go analysis of JDF12 on DU145 cells. [file 8050313.f1.pdf]

# Supplementary materials

# Potential Anticancer Mechanisms of a Novel EGFR/DNA-targeting Combi-molecule (JDF12) against DU145 Prostate Cancer Cells: An iTRAQ-based Proteomic Analysis

**Haofeng Zheng[1a], Guancan Liang[1a], Yanxiong Chen[1], Sijie Lin[1], Wei Liu[2], Youqiang Fang[1*]**

[1]Department of Urology, the Third Affiliated Hospital of Sun Yat-Sen University, Guangzhou, 510630, China.

[2]Guangdong Provincial Key Laboratory of Liver Disease Research, the Third Affiliated Hospital of Sun Yat-Sen University, Guangzhou, 510630, China.

***Corresponding author.**

Department of Urology, The Third Affiliated Hospital of Sun Yat-sen University, 600W Tianhe Road, Guangzhou 510630, China.

Tel. +86 20 85252052; Fax: +86 20 85252678.

Email: fangyq@mail.sysu.edu.cn .

[a] These authors contributed to this paper equally.

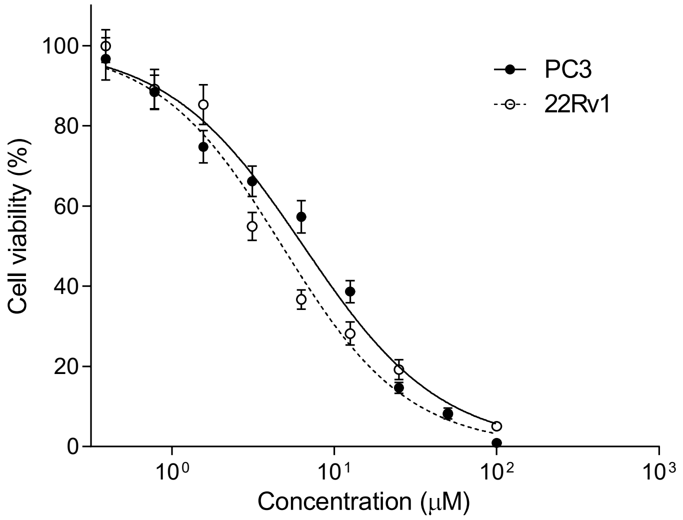


**Supplemental Figure 1** The effects of JDF12 on the cell viability for PC3 and 22Rv1 cell lines at 48h. $IC_{50}$ for PC3 is 6.45 ±0.43μM, and for 22Rv1 is 4.77±0.28μM. Data was expressed as mean ± SD of 5 determinations from three independent experiments.

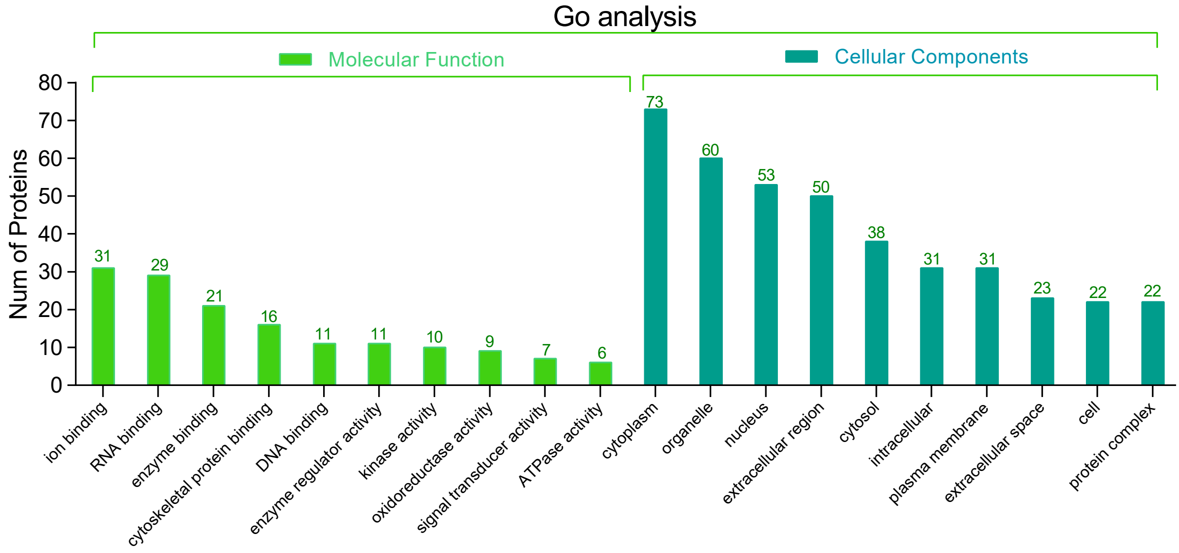


**Supplemental Figure 2** The functional classification of differentially expressed proteins using Go analysis (Molecular Function and Cellular Components views)
